# Anterior-First Approach for Latissimus Dorsi Myocutaneous Flap Breast Reconstruction: A Refined Elevation Method with Detailed Video Instructions

**DOI:** 10.3390/jcm11247387

**Published:** 2022-12-13

**Authors:** Jangyoun Choi, Eun Jeong Ko, Sung Ae Kim, Jong Yun Choi, Suk-Ho Moon, Young Joon Jun, Jun Hee Byeon, Deuk Young Oh

**Affiliations:** 1 Department of Plastic and Reconstructive Surgery, Seoul St. Mary’s Hospital, College of Medicine, The Catholic University of Korea, Seoul 06591, Republic of Korea; 2 Department of Plastic and Reconstructive Surgery, Eunpyeong St. Mary’s Hospital, College of Medicine, The Catholic University of Korea, Seoul 03312, Republic of Korea

**Keywords:** breast cancer, breast reconstruction, Latissimus Dorsi, autologous, surgical procedure

## Abstract

Background: The latissimus dorsi myocutaneous (LDMC) flap is a preferred flap in breast reconstruction for its wide surface area and volume. Since the flap is situated in the midback area, a lateral decubitus approach is a conventional method. However, proper visualization and access to the thoracodorsal vascular pedicle or muscle insertion is difficult from the lateral approach, causing inefficiency and surgeon fatigue. We propose the ‘anterior-first’ approach in LDMC flap reconstruction, where the landmark structures are first approached from the supine-anterior position through the mastectomy incision. Methods: From January 2014 to December 2020, 48 patients who received immediate breast reconstruction with LDMC flap were included in the study. Patients received reconstruction with the conventional approach (*n* = 20), or anterior-first approach (*n* = 28). Demographic factors and the operative outcomes were retrospectively analyzed and compared between the two groups. Results: Compared to the conventional approach group, the anterior-first approach group showed improved efficiency in the duration of total reconstruction (228 versus 330 min, *p* < 0.001), and flap elevation (139 versus 200 min, *p* < 0.001). No difference in complication rate and time to drain removal was observed (*p* = 0.14 and >0.9, respectively). Conclusion: The anterior-first approach for breast reconstruction with LDMC flap provides surgeons with an enhanced surgical exposure and superior ergonomics, leading to a safer and more efficient flap elevation.

## 1. Introduction

The Latissimus Dorsi myocutaneous (LDMC) flap is a workhorse flap in the reconstructive armamentarium for breast reconstruction [1]. After its first use in chest wall reconstruction, the LDMC flap was popularized for coverage of postmastectomy defects [2,3,4,5,6]. When reconstructing a total mastectomy defect, a transverse-to-diagonal flap design over the midback area is usually planned for maximal volume recruitment [7]. With this design, the flap elevation usually starts from the patient positioned in a decubitus position. However, inefficiency in flap dissection arises in performing dissection of the cranial-axillary portions of the flap. This is mainly due to poor visualization of the surgical field and awkward surgeon ergonomics. Improving this portion of the procedure is highly beneficial because critical structures related to flap perfusion and transfer lie in this area. In this article, we would like to share our refined method of flap elevation by using the anterior-first approach for breast reconstruction with LDMC flap.

## 2. Materials and Methods

### 2.1. Ethics Statement and Patient Selection

This retrospective study was approved by the Institutional Review Board of Catholic Medical Center (IRB No. KC22RASI0500) and was conducted following the Declaration of Helsinki. Retrospective medical data were collected for patients who underwent immediate breast reconstruction with extended LDMC flap, with a conventional (lateral) approach, or the anterior-first approach methods from January 2014 to December 2020. Delayed, bilateral, and simultaneous implant reconstruction cases were excluded. Patient demographics are summarized in Table 1.

### 2.2. Study Design

Through retrospective chart review, we gathered demographics, oncologic data, reconstructive parameters, and postoperative outcomes of the conventional approach group and the anterior-first approach group. Oncologic data included information about chemotherapy, type of mastectomy, presence of axillary lymph node dissection, and weight of mastectomy. Reconstructive parameters included flap dimensions (surface area) and cumulative operative time of each phase of the reconstructive surgery. Postoperative data included postoperative drainage, time to discharge, and complications (flap necrosis, wound infection, seroma). From March 2010 to December 2015, the conventional approach was implemented in all cases. From 2016 to 2020, the anterior-first approach was implemented in all cases. All reconstruction was performed by the same team.

### 2.3. Statistical Analysis

Data analyses were performed using the R statistical software (R Foundation for Statistical Computing, Vienna, Austria) [8]. Continuous variables were analyzed with Mann–Whitney U test. Discrete variables were analyzed using the chi-square test or fisher’s exact test to determine their significance between the two groups. Data visualization was performed with R package ggplot2 (R Foundation for Statistical Computing, Vienna, Austria) [9].

### 2.4. Operative Procedure

After mastectomy, the brief steps for breast reconstruction with the LD flap can be divided into three blocks, which are (1) flap elevation; (2) position change; and (3) flap inset. Our method separates the flap elevation block into two subphases, of which the first subphase is performed before the first position change (Table 2).

**Figure 1:**
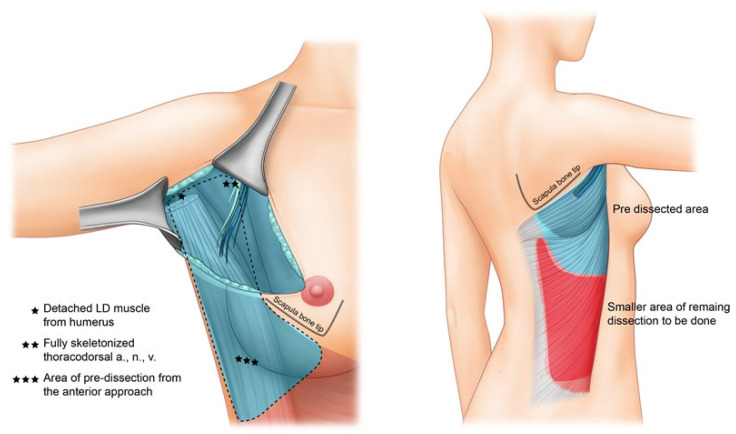
Brief graphical presentation of the proposed method.

Following mastectomy and within the same field, the first subphase of the flap elevation is undertaken. The axillary and the lateral portion of the LD muscle body, the humeral attachment of LD muscle, and the neurovascular pedicle of the LD muscle is clearly delineated and skeletonized. Posterolateral undermining of the trunk skin and tunnel formation are performed in this first subphase of the flap elevation, directly through the mastectomy incision in supine position. (Figure 1)

**Figure 2:**
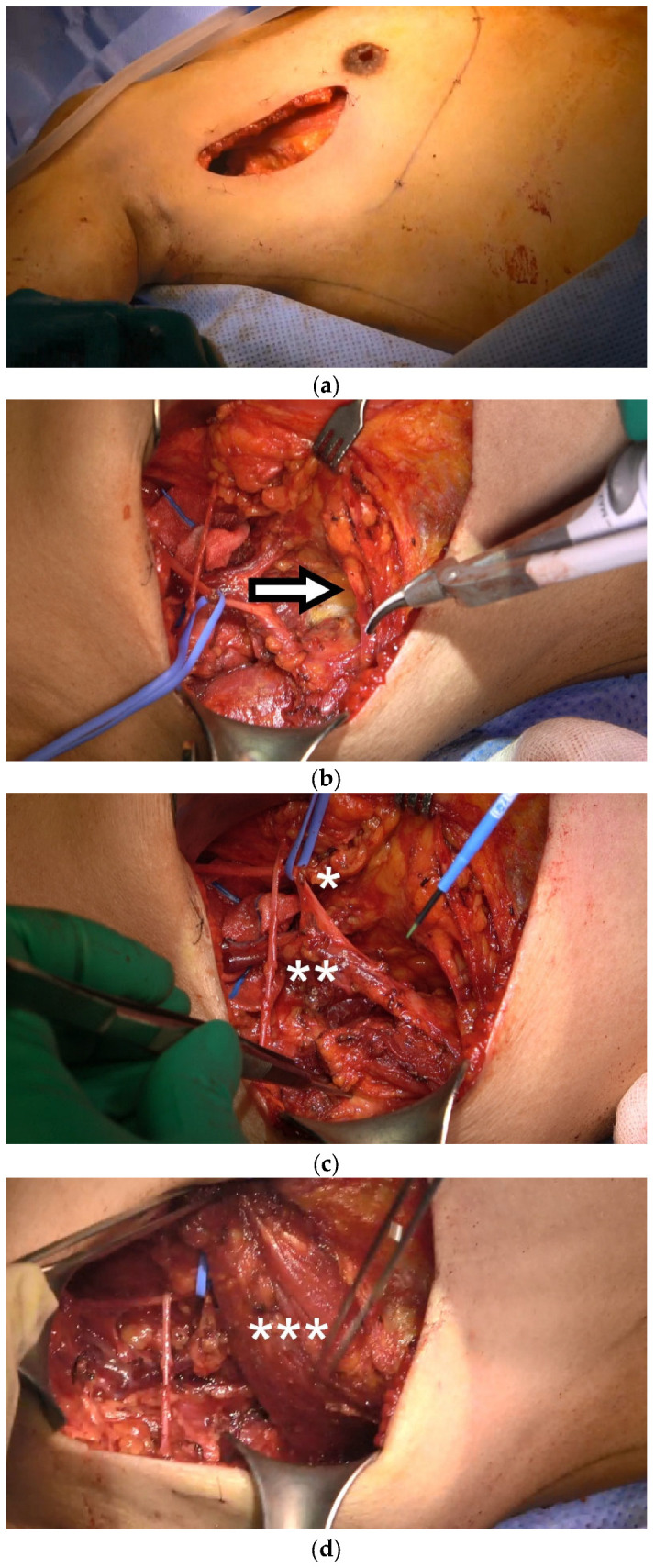

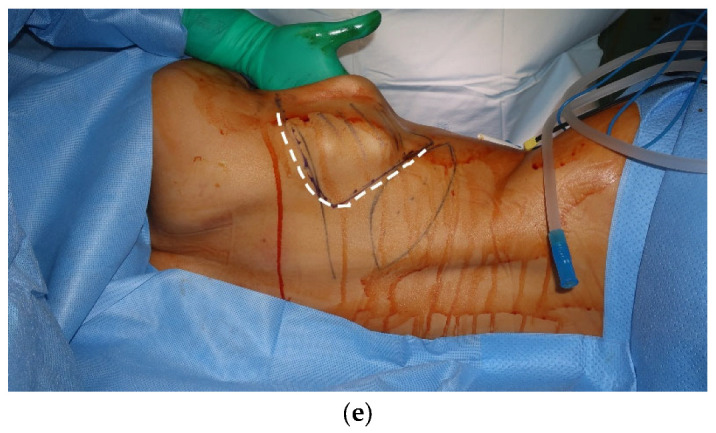
Operative procedure. (**a**) Anterior approach is performed in the supine position, using the mastectomy incision; (**b**) plane between the LD–serratus interface is developed (arrow); (**c**) complete skeletonization of thoracodorsal nerve (single asterisk), artery and vein (double asterisk) is convenient and can be performed in an efficient manner with great surgeon ergonomics; (**d**) the humeral insertion of LD muscle (triple asterisk) is detached; (**e**) a substantial amount of pre-tunneling and dissection can be performed (dashed line).

A detailed video instruction can be found in the Supplementary Materials (Supplementary Video S1). Through the mastectomy field, we first identify the lateral border of the LD muscle and develop a plane between the LD–serratus interface (Figure 2a,b). The long thoracic neurovascular pedicle is preserved. Deepening the dissection, the thoracodorsal pedicle is visible and care is taken to preserve its intramuscular entry. Usually, the thoracodorsal nerve is more superficial and thicker in diameter, and this serves as a visual guide in avoiding injury to the thoracodorsal artery and vein. Complete isolation of the thoracodorsal vascular pedicle from the surrounding soft tissues is (Figure 2c). One can easily achieve a long length of vascular pedicle up to the subscapular branching point of the axillary artery. After complete preservation of the thoracodorsal neurovascular bundle, the attachment of LD muscle to the humerus is identified. Following the fibers of the LD muscle, sufficient dissection in a cranial direction towards the shoulder joint reveals the white tendinous portion of the LD muscle. The tendinous portion is disinserted and completely freed from the humerus (Figure 2d). Subfascial elevation of the lateral trunk skin from the LD muscle is performed as far posterior as possible. With this procedure, approximately one-third of the LD muscle can be mobilized from the anterior approach only, before turning the patient to the lateral decubitus position. After sufficient undermining and muscle mobilization, the patient is turned into a decubitus position to elevate the remaining flap territory from the back in an expeditious manner (Figure 2e).

After changing the position of the patient to lateral decubitus, the remaining portion of the flap elevation is performed. The pre-formed space created by subcutaneous undermining from the anterior approach can be easily reached through a brief dissection of few centimeters from the skin paddle margin. A standard supra-fascial dissection is done to fully outline the midline and lower borders of the LD muscle. Sub-LD plane is developed from the inferior edge of the LD muscle and carried out in a superolateral dissection towards the axilla. This dissection is also easily connected with the predissected sub-LD plane that was developed from the anterior approach.

## 3. Results

### 3.1. Demographic and Oncologic Data

A total of 48 patients were recruited for the study. Overall, 28 patients received LDMC flap breast reconstruction with the anterior-first approach. In total, 20 patients received the reconstruction with the conventional approach. There was no statistical difference in demographic data between the two groups. A trend was seen favoring nipple-sparing mastectomy for the type of mastectomy in the anterior first approach group (Table 1, *p <* 0.001).

### 3.2. Operative Outcomes Data

**Figure 3:**
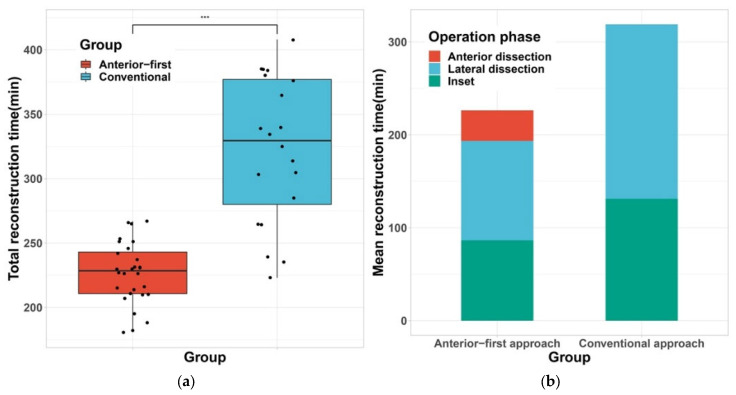
Comparison of operative time between two groups. (**a**) Boxplot of total reconstruction time shows a shortened operative duration in the Anterior-first approach group; (**b**) Stacked bar plot of mean reconstruction time of each surgical sequence shows shorter flap elevation time (red plus blue), and inset (green), *** denotes for *p* < 0.001.

Compared to the conventional approach group, the anterior-first approach group showed a shorter operative time (*p <* 0.001, Table 2 and Figure 3a). The mean operative time of each subphase showed a shorter duration in the anterior-first group (*p* < 0.001, Figure 3b). Most notably, flap elevation time showed a significant decrease in the anterior-first group (139 versus 180 min, Table 3). Low rates of reversible complications such as hematoma, nipple color change, or seroma were shown in both groups (*p =* 0.14, Table 3). Days to drain removal and flap size did not show difference with statistical significance between two groups (Table 3).

**Figure 4:**
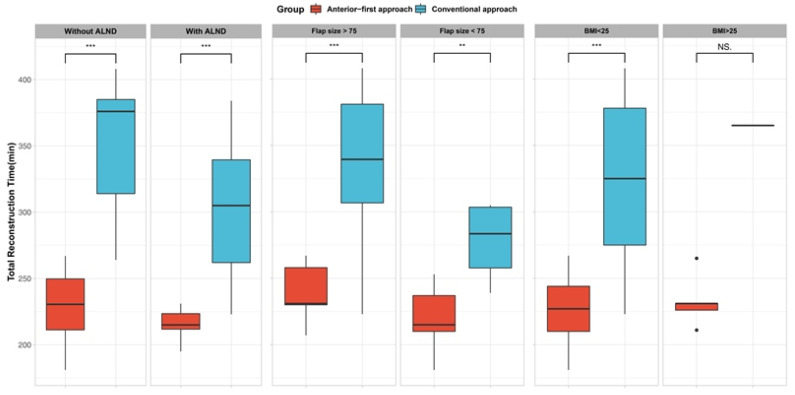
Sub-group analysis by potential confounding variables. Anterior-first approach shows statistically significant reduction in total reconstruction time, regardless of axillary lymph node dissection (ALND) status, flap size. However, Level of BMI failed to show significance. ** denotes for *p* < 0.01, *** denotes for *p* < 0.001, NS, not significant.

We also performed grouped analysis under factors that may affect the operative duration (Figure 4). Overall, the anterior-first approach exhibited shorter operative times, regardless of axillary clearance or a large flap size. However, in high BMI patients, the anterior-first approach failed to show significant reduction in operative duration.

## 4. Discussion

Since the first introduction of its use for breast reconstruction in 1970, the LDMC flap still holds an important role in the reconstruction of small to medium-sized breasts for postmastectomy defects [7,10]. However, the problem of time efficiency in a classic LDMC flap design has room for improvement [11]. In our study, we proposed a refined method of elevating the anterior portion of the flap, which corresponds to the axillary and lateral regions of the trunk, in the mastectomy position (i.e., supine position) before turning the patient to a decubitus position. In the conventional lateral approach, the thoracodorsal pedicle is hidden in the axillary undersurface of the LD muscle. Therefore, direct visualization of the pedicle is impossible and hinders a safe and confident dissection. Our method enables the surgeon to perform the most critical steps of the flap elevation process under direct vision, with enhanced surgeon ergonomics. On analysis, our results showed that this change in flap elevation sequence may enhance operative efficiency, and thus reduce operation time.

Previous studies that claim their refined methods of LDMC flap elevation usually gained efficiency by modifying the orientation of the skin paddle and limiting the flap dissection to obtain a smaller volume [12]. As a tradeoff between a quicker flap harvest, major renunciation arises in flap volume, which is a critical factor when considering autologous-only breast reconstruction. For example, elevating the LDMC flap from a purely supine position without position change may be the quickest manner to perform an LDMC flap. However, this approach restricts the skin paddle location and design into a lateralized, and vertical orientation along the posterior axillary line. Therefore, this lateralized, vertically oriented flap design is only indicated in select cases requiring a very small volume such as partial breast reconstruction [12,13,14]. Therefore, previous studies were able to achieve faster flap elevation only in exchange with smaller flap volume and vertical donor scar, which in majority of cases required additional implant placement or fat graft [13,15,16,17]. In contrast, our study proposes a refinement that can be applied in a conventional design of LDMC flap that recruit most of the back tissue with the horizontal skin paddle in the bra line.

Our refinement may look counterintuitive because it seems that an additional step is added to the original sequence of flap elevation. However, there is no additional procedure added to the conventional flap elevation. The essentials of refinement lie in the dissecting the axillary area first, in the supine position. With this change in patient position and order of dissection, our results show that the operative time was greatly reduced by 31 percent (330 to 228 min, *p <* 0.001). We think that this efficiency is directly related to the surgeon’s ergonomics and a wider surgical field that is provided with the anterior-first approach. Complete detachment of the humeral insertion of LD muscle, and complete skeletonization of the thoracodorsal neurovascular bundle is the core procedures in flap elevation. These two core procedures are decisive factors in a successful breast reconstruction with LDMC flap. Performing detachment of the LD muscle and isolation of the thoracodorsal pedicle from the back area is tedious because the anatomical structures are deeply seated in the axillary fossa, in the undersurface of LD muscle. In the anterior-first approach, these procedures can be done with straightforward visualization and ergonomics which leads to an overall safe and efficient procedure.

Breaking down the operative steps into smaller subsequences, we were also able to see 42 min reduction in the inset and closure times in the anterior-first approach group. Musculocutaneous pedicled flap reconstruction such as LDMC flap requires maximal mobilization of the flap for an optimal inset, because gaining a maximal arc of rotation allows a higher degree of freedom in flap movement and facilitates a faster and aesthetic breast mound shaping. In that aspect, we believe that shorter inset times may also have originated from the surgeon’s convenience in gaining maximal arc of rotation, which was made possible by first performing complete skeletonization of the thoracodorsal pedicle up to the axillary artery, and fully detaching the LD muscle from the humerus in the anterior-first approach.

Our method can be implemented in other reconstructive demands. Oncologic or traumatic defect to the sternum or chest wall pose similar reconstructive demand as the postmastectomy defect, and LDMC flap reconstruction is a popular choice [18]. Since sternal and chest wall defects are presented to the reconstructive surgeon in the supine position, the anterior-first approach can be utilized in the same fashion to aid in convenient and safer reconstruction. Using the anterior-first approach can benefit the most when the size of the flap skin paddle is small. A small skin paddle can only provide a deep and narrow surgical field when the surgeon is approaching the axillary area from the back. However, the anterior-first approach is not affected by the small skin paddle size because the length of the lateral mastectomy incision ensures wide and direct exposure to the axillary contents. However, the extent of anterior dissection can be limited in patients with thin and tight-skinned patients, or highly obese patients due to their innate tissue character. Extremely lean or obese patients may have lesser benefit from anterior dissection because of tight skin or narrow window due to abundant soft tissue. This may in part explain the result in sub-group analysis, which did not show a significant benefit of anterior dissection in patients with BMI > 25.

Several limitations of this study must be acknowledged. Cohort size is limited, thus limiting statistical power. Therefore, more cases are required. There might have been an institutional bias since the surgery was performed by the same team. Since the study was retrospective in nature, no randomization was performed. If a distant incision such as periareolar, inframammary incision is used, the anterior-first technique is not indicated.

## 5. Conclusions

Although accepted as one of the gold-standard solutions for breast reconstruction, LDMC flaps possess intrinsic drawbacks in terms of surgical efficiency. Performing the vascular pedicle isolation and muscular disinsertion in the supine position, rather than the conventionally performed lateral position, greatly enhances the surgical field, and benefits the surgeon’s ergonomics. This leads to a reduced operative time and a more efficient flap inset.

## Figures and Tables

**Table 1:** Patient demographics.

Characteristic	Anterior-First Approach, *n* = 28 ^1^	Conventional Approach, *n* = 20 ^1^	*p*-Value ^2^
Age	45 (40, 51)	42 (38, 48)	0.2
Type of mastectomy			<0.001
MRM	1 (3.6%)	7 (35%)	
NSM	14 (50%)	1 (5.0%)	
SSM	13 (46%)	12 (60%)	
BMI	21.8 (20.9, 24.2)	20.0 (19.1, 21.3)	0.004
DM	1 (3.6%)	0 (0%)	>0.9
Smoking	0 (0%)	0 (0%)	
Adjuvant chemotherapy	3 (11%)	2 (10%)	>0.9
Axillary node dissection	6 (21%)	11 (55%)	0.017
Mastectomy weight	247 (161, 341)	235 (150, 303)	0.8
Flap surface area	68 (51, 75)	82 (77, 93)	0.010

^1^ Median (IQR); *n* (%); ^2^ Wilcoxon rank sum exact test; Wilcoxon rank sum test; Fisher’s exact test; Pearson’s Chi-squared test; MRM: modified radical mastectomy; NSM: nipple-sparing mastectomy; SSM: skin sparing mastectomy; BMI: body mass index; DM: diabetes mellitus.

**Table 2:** A brief comparison of the surgical steps undertaken using the anterior-first approach and the conventional approach.

Operative Phase	Anterior-First Approach	Conventional Approach
Flap elevation	1st sub-phase: Elevation of axillary and lateral portion of LDMC flap without position change	N/A
Position change	Position change (supine → lateral)	Position change (supine → lateral)
Flap elevation	2nd sub-phase: Elevate remaining distal area of the flap	Initiation of flap elevation from the lateral position
Position change	Position change (lateral → supine)	Position change (lateral → supine)
Inset and Closure	Flap inset and closure	Flap inset and closure

**Table 3:** Comparison of operative duration and surgical outcomes.

Characteristic	Anterior-First Approach, *n* = 28 ^1^	Conventional Approach, *n* = 20 ^1^	*p*-Value ^2^
Flap elevation (minutes)	139 (129, 145)	200 (165, 228)	<0.001
Anterior dissection	33 (29, 37)	0 (0, 0)	
Lateral dissection	104 (98, 116)	200 (165, 228)	<0.001
Inset and closure (minutes)	82 (71, 100)	124 (100, 150)	<0.001
Total reconstruction time (minutes)	228 (211, 243)	330 (280, 377)	<0.001
Complication (%)			0.14
Hematoma	1 (3.6%)	1 (5.0%)	
Nipple color change	0 (0%)	1 (5.0%)	
Seroma	0 (0%)	2 (10%)	
Drain removal (days)	7.0 (5.0, 12.2)	8.0 (6.0, 10.0)	>0.9
Flap size (cm^2^)	68 (51, 75)	82 (77, 93)	0.010

^1^ *n* (%); Median (IQR); ^2^ Fisher’s exact test; Wilcoxon rank sum test.

## Data Availability

The data presented in this study are available on request from the corresponding author.

## Supplementary Materials

The following supporting information can be downloaded at: https://www.mdpi.com/article/10.3390/jcm11247387/s1, Video S1: Video vignette: Anterior-first approach Latissimus Dorsi Musculocutaneous flap in Breast Reconstruction.
